# Effect of storage time on biochemical characteristics and antioxidant activity of hawk tea (*Litsea coreana*) processed by boiling water fixation

**DOI:** 10.1002/fsn3.1913

**Published:** 2020-10-07

**Authors:** Qing Xu, Yuanjing Zhou, Jingfang Zhao, Songlin Yao, Jihong Wang

**Affiliations:** ^1^ Institute of Biology Guizhou Academy of Sciences Guiyang City China; ^2^ Guizhou Academy of Analysis and Testing Guizhou Academy of Sciences Guiyang City China; ^3^ Guizhou Institute of Mountain Resources Guizhou Academy of Sciences Guiyang City China

**Keywords:** antioxidant activity, biochemical characteristics, Hawk tea, storage time

## Abstract

This study investigated the effect of storage time on biochemical characteristics of hawk tea (*Litsea coreana*) and explored the correlation between the content of flavonoids and polyphenols and antioxidant activity. The antioxidant activity and the content of inclusions, amino acid, and mineral elements in hawk tea processed by boiling water fixation and packed in airtight polypropylene bags and stored in 0°C refrigerator under different storage time (one year, three years, and six years) were analyzed. Results indicated that the biochemical characteristics of hawk tea changed less within 12 months. The total content and types of amino acids in hawk tea reached the maximum in the third year, as well as the content of total trace elements. The water extracts, tea polyphenol, caffeine, lysine, valine, isoleucine, glycine, proline, Ca, and Zn decreased continuously in the storage period of 6 years, but the total flavonoids, Mg, and Ni changed just the opposite. Total polyphenol is the main antioxidant material in hawk tea. Results of the present study provided useful information for people to systematically understand the changes of tea in the storage process and to reasonably develop hawk tea product.

## Graphical abstract

The biochemical characteristics of hawk tea processed by boiling water fixation changed less within 12 months. The total content and types of amino acids in hawk tea reached the maximum in the third year, as well as the content of total trace elements. The water extracts, tea polyphenol, caffeine, lysine, valine, isoleucine, glycine, proline, Ca, and Zn decreased continuously in the storage period of 6 years, but the total flavonoids, Mg, and Ni changed just the opposite. Total polyphenol is the main antioxidant material in hawk tea.

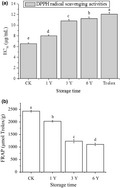

## 1 INTRODUCTION

Hawk tea (*Litsea coreana*) is a traditional folk beverage and traditional Chinese medicine (Jia and Yuan, 2019). As a traditional heat‐relieving beverage in the hot and humid areas of Chishui River Basin, China, hawk tea can relieve heat and thirst and alleviate distention (Xi et al., 2015). It also has antidiabetic (Lu et al., 2010), hypolipidemic (Zhao, 2013), antioxidant (Chen et al., 2019), antimicrobial (Yu et al., 2016), and hepatoprotective activity (Jia and Yuan, 2019) as well as anti‐inflammatory properties (Liu et al., 2020).

Hawk tea tree is a woody plant of Lauraceae, belonging to the tropical species of East Asia, mainly distributed in China, and may also be distributed in Korea or Japan, but it is rarely reported. As an undergrowth crop in forests, hawk tea tree is widely cultivated in southwestern China, particularly in Guizhou Province where it is a major agroforestry crop (Wang et al., 2010). According to the processed material (buds or leaves) of *L. coreana* var. *lanuginose*, hawk tea is divided into bud hawk tea (buds) and hawk tea (tender and mature leaves). Hawk tea is traditionally processed by picking the buds and new leaves in middle and late April and drying them slowly after fixing in an iron pot. But in Xifeng county and Chishui city, Guizhou province, the newly mature leaves are picked in early May and then dried slowly after fixing in boiling water. This is used to feed the larvae of *Aglossa dimidiate* or *Hydrillodes morosa,* and the feces of the larvae are commonly known as insect hawk tea or sandy tea (Jia et al., 2017).

Hawk tea is abundant in proteins, amino acids, sugars, polyphenols, and flavonoids, which are closely related to its physiological activity, and varies with the processing methods (Huang et al., 2016; Park, 2017; Xu, et al. 2017; Xu, et al., 2016). It had been devoted more attention to the extraction and health benefits of flavonoids, polysaccharides, and essential oils from new hawk tea in recent years (Jia et al., 2014, 2017; Qin et al., 2018). The contents of total flavonoids and total polyphenol in hawk tea were varied with the seasons (Xiao et al., 2015), and the contents of trace elements (Pb, Cd, Mn, Fe, Zn, and Ca) in hawk tea from different areas were varied (Gu and Peng, 2013). However, less attention has been paid to the research on the change of chemical constituents in hawk tea with storage time. This paper intended to explore the effect of storage time on biochemical characteristics of hawk tea processed by boiling water fixation. The variation of biochemical characteristics such as the inclusions, amino acids, and mineral elements of the hawk tea with storage for 0, 1, 3, and 6 year was analyzed systematically for the first time. The correlation between the content of flavonoids and polyphenols and antioxidant activity was explored. These results would provide scientific basis for commercial utilization of hawk tea.

## 2 MATERIALS AND METHODS

### 2.1 Materials

Tender leaves (one bud and five to six leaves) from wild *L. coreana* var. *lanuginose* plants growing in Xifeng County, Guizhou Province, China, were collected in April 2014. They were processed by the Guizhou Institute of Tea Science on the same day according to the method of tea fixation by boiling water. The teas were packed in airtight polypropylene bags and stored in 0°C refrigerator until further use.

### 2.2 Methods

#### 2.2.1 Tea fixation by boiling water

**FIGURE 1:**
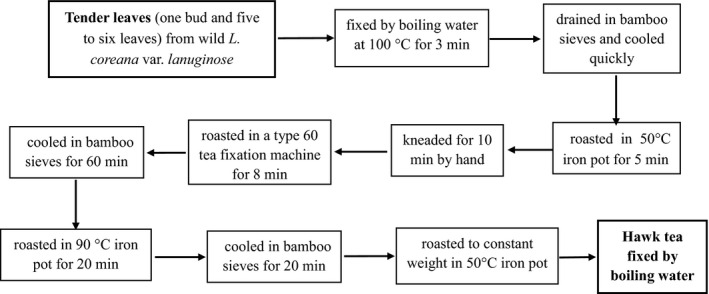
Process flow chart of preparing hawk tea fixation by boiling water

The process flow chart of preparing hawk tea fixation by boiling water was shown in Figure 1. Go through 9 steps to obtain hawk tea processed by boiling water fixation.

#### 2.2.2 Biochemical quality test

Determination of water extracts in the hawk tea was according to the National Standard of the People's Republic of China GB/T 8305–2013 “Tea determination of water extracts content.” Two grams of dry and ground sample was weighed and placed into a 500‐ml conical flask, to which 300 ml of boiling distilled water was added, following which the mixture was immediately transferred to a boiling water bath for 45 min (with shaking every 10 min). Following the extraction, the tea residue was filtered immediately under hot decompression and washed several times with about 150 ml of boiling distilled water. The tea residue and a known quality of filter paper were then placed into a baking dish and transferred to a constant temperature drying oven at 120°C ± 2°C for 1 hr. Following this, the tea was cooled for 1 hr, baked for 1 hr, immediately transferred to a dryer in order to cool to room temperature, and then weighed. The content of water extract in the hawk tea was calculated according to formula (1):

(1) $$\text{Content of water extract (}\%)=[\left(1‐\left(m_{1}/m_{0}\right)\right]\times 100\%,$$

where m_0_ is the mass of the dry sample, g; and m_1_ is the mass of the dried tea residue, g.

Determination of soluble sugar in the hawk tea was according to the National Standard of the People's Republic of China GB/T 8305–2013 “Method for analysis of forestry biomass‐Determination of soluble saccharides content.” a) The glucose standard curve was prepared to evaluate the soluble sugar in terms of glucose equivalents. Ten milligrams of glucose and deionized water was mixed and diluted to concentrations of 5, 10, 15, 20, 25, or 30 μg/ml. b) To prepare the test solution, 0.2 g of uniformly ground dry tea sample was weighed into a 10‐ml centrifuge tube, to which 5 ml of 70% methanol solution (preheated at 70°C) was added, following which the mixture was stirred and wet evenly and immediately transferred to a 70°C water bath and extracted for 10 min (with stirring every 5 min). The mixture was then cooled to room temperature and centrifuged at 3,500 r/min for 10 min, and then, the supernatant was transferred to a 10‐ml volumetric flask. The residue was extracted twice with 5 ml of 70% methanol solution, and the above operation was repeated. The extraction solution was combined to make 15 ml, shaken well, and passed through a 0.45‐µm membrane. Two milliliters was transferred to a 10‐ml volumetric flask with a pipette. 3) For the determination, a pipette was used to transfer 1 ml of glucose working fluid, water, and measurement solution, respectively, to a 10‐ml graduated tube, to which 3 ml of anthrone reagent was added. The mixture was kept at 90°C for 15 min and then cooled, following which the absorbance value was measured at 620 nm using a UV/Vis spectrophotometer (Schimadzu, Japan) to calculate the total soluble sugar content using the glucose standard curve.

The protein content in the hawk tea was determined by spectrophotometry according to the National Food Safety Standard GB/T 36056–2018 “Determination of protein in foods” using the ammonia nitrogen standard curve. 1) An ammonia nitrogen standard curve was prepared to evaluate the protein in terms of the standard series and the conversion coefficient. Ammonium sulfate (0.4720 g) was dissolved in water and made up to 100 ml in a volumetric flask. Ten milliliters was pipetted into a 100‐mL volumetric flask with water added to make up the volume and then mixed well. Each mL of this solution is equivalent to 0.1 mg nitrogen. We then pipetted 0.00 ml, 0.05 ml, 0.10 ml, 0.20 ml, 0.40 ml, 0.60 ml, 0.80 ml, and 1.00 ml ammonia nitrogen standard solution (equivalent to 0.00 μg, 5.00 μg, 10.0 μg, 20.0 μg, 40.0 μg, 60.0 μg, 80.0 μg, and 100.0 μg nitrogen) into 10‐ml colorimetric tubes, respectively. Four milliliters of sodium acetate–acetic acid buffer solution and 4.0 ml chromogenic reagent (15 ml of formaldehyde with 7.8 ml of acetylacetone, diluted with water to 100 ml, vigorously mixed and placed at room temperature for 3 d) were combined with water to scale and mixed well. The mixture was heated in a 100°C water bath for 15 min. Once it had cooled to room temperature, it was transferred into a 1‐cm cuvette and the absorbance at 400 nm was measured, using a blank tube as the reference. The standard curve was drawn according to the absorbance value. 2) The test solution was prepared by transferring 0.1 g–0.5 g tea sample into a dry 100‐ml Kjeldahl flask, to which 0.1 g copper sulfate, 1 g potassium sulfate, and 5 ml sulfuric acid were added. The mixture was slowly heated until the contents were carbonized and foaming, and the temperature was increased to keep the liquid in the bottle boiling slightly until the liquid become blue‐green and transparent, following which it was heated for 0.5 hr. After cooling, 20 ml of water was slowly added and the mixture was transferred into a 50‐ml volumetric flask. The Kjeldahl flask was mixed with a small amount of water, following which water was added to scale and mixed well. Two to five milliliters was pipetted into a 50‐ml volumetric flask, to which 1–2 drops of p‐nitrophenol indicator solution was added. The mixture was shaken well, and sodium hydroxide solution was added to neutralize it to yellow, following which acetic acid solution was added to make the solution colorless. The mixture was then diluted to scale with water and mixed well. 3) For determination, 0.5–2 ml (about equal to nitrogen < 100 μg) of sample solution and the same amount of reagent blank solution were, respectively, pipetted into 10‐ml colorimetric tubes. The absorbance at 400 nm was measured in the same way as the standard curve above, and the protein content was measured in the hawk tea by using the ammonia nitrogen standard curve.

The fat content of the hawk tea was determined by using the Soxhlet method according to the National Food Safety Standard GB/T5009.5—2003 “Determination of fat in foods.” Dried tea leaves were pulverized through a 40‐mesh sieve with a pulverizer. Two to five grams was placed into a filter paper tube and then placed into the extraction tube of a fat extractor, to which petroleum ether was added (boiling range 30–60°C), heated to reflux (6–8 times/h), and extracted for 6–12 hr. The receiving bottle was removed, and the mixture was concentrated under reduced pressure to remove the petroleum ether, dried for 2 hr at 100°C ± 5°C, placed into the dryer for cooling, and weighed. The content of fat in the hawk tea was calculated according to formula (2):

(2) $$\text{Content of crude fat (}g/l00g)=\left[\left(m_{1}‐m_{0}\right)/m_{2}\right]\times 100,$$

The total ash in the hawk tea was determined according to the National Food Safety Standard GB 5,009.4–2010 “Determination of ash in foods.” a) A porcelain crucible of an appropriate size was placed in a muffle furnace for 0.5 hr at 550 ± 25°C, following which it was cooled to about 200°C, removed, and placed in a desiccator and cooled for 30 min and then weighed accurately. The burning process was repeated until the weight difference was less than 0.5 mg. b) Two to three grams of sample was used for those with ash contents greater than 10 g/100 g, while 3 g to 10 g was weighed for samples with ash contents less than 10 g/100 g. 3) For determination, the sample was first heated on an electric heating plate with a small fire to fully carbonize it until there was no smoke, following which it was placed in a muffle furnace and burned for 4 hr at 550 ± 25°C. It was then cooled to about 200°C, removed, and placed into a desiccator for 30 min. If carbon particles were present in the burned residue before weighing, a little water was dropped into the sample to moisten it, the water was evaporated, and the sample was burned until there were no visible carbon particles. The burning process was repeated until the weight difference was less than 0.5 mg. The following formula was used (3):

(3) $$\text{Content of total ash (}\%)=\left[\left(m_{1}‐m_{2}\right)/\left(m_{3}‐m_{2}\right)\right]\;\times 100\%,$$

where m_1_ is the mass of the crucible and ash, g; m_2_ is the mass of the crucible, g; and m_3_ is the mass of crucible and sample, g.

Determination of total flavonoids in the hawk tea used the sodium nitrite‐aluminum nitrate‐sodium hydroxide color system as described by Xiao et al. (2015) with some modifications. A rutin standard curve was prepared to evaluate the total flavonoids in terms of rutin equivalents. Six milligrams of rutin and 70% methanol solution were mixed and diluted in concentrations of 1.2, 2.4, 3.6, 4.8, 6.0, or 7.2 μg/ml. 2) The test solution was prepared using the same step as the above soluble sugar. 3) For determination, 1 ml of rutin working fluid, water, and measurement solution were, respectively, pipetted into a 10‐ml graduated tube to which 1 ml of sodium nitrite (5%) was added, shaken well, and allowed to stand for 6 min. One milliliter of 10% aluminum nitrate was added, shaken well, and allowed to stand for 6 min. Four milliliters of 4% sodium hydroxide was added. The total volume of the mixture was made to 10 ml by adding 70% methanol, and the tubes were vigorously stirred. The resulting solution was pink, and its absorbance was determined using a spectrophotometer at 510 nm against a 70% methanol. The content of total flavonoids in the hawk tea was calculated using the rutin standard curve.

Determination of total polyphenols in the hawk tea using the Folin–Ciocalteu reagent method as described by Xiao et al. (2015) and the National Standard of the People's Republic of China GB/T8313‐2008 “Determination of total polyphenols and catechins content in tea.” a) The gallic acid standard curve was prepared to evaluate the total phenolics in terms of gallic acid equivalents. Ten milligrams of gallic acid and deionized water was mixed and diluted in concentrations of 5, 10, 15, 20, 25, or 30 μg/ml. b) The test solution was prepared using the same step as the above soluble sugar. c) For determination, a pipette was used to transfer 1 ml of gallic acid working fluid, water, and measurement solution, respectively, to a 10‐ml graduated tube, to which 5 ml of Folin–Ciocalteu reagent (10%) was added and shaken well. After reacting for 3–8 min, 4 ml of 7.5% Na_2_CO_3_ solution was added, topped up with water to 10 ml, and shaken well. The mixture was allowed to stand at room temperature for 60 min, following which the absorbance was measured at 765 nm using distilled water as a blank. The content of total polyphenols in the hawk tea was calculated using the gallic acid standard curve.

The caffeine content was determined by ultraviolet spectrophotometry according to the National Standard of the People's Republic of China GB/T 8312–2013 “Tea determination of caffeine content.” a) A caffeine standard curve was prepared to evaluate the caffeine in terms of caffeine equivalents. Ten milligrams of caffeine and deionized water was mixed and diluted to concentrations of 5, 10, 15, 20, 25, or 30 μg/ml. One milliliter of caffeine working fluid was transferred to a 25‐ml volumetric flask, to which 1.0 ml of 0.01 mol/L hydrochloric acid was added and diluted to scale with distilled water and mixed well. Distilled water was used as a blank reference, the absorbance was measured at 274 nm, and a curve between the absorbance and the corresponding caffeine concentration was generated. b) The test solution was prepared using the same step as the above water extract. c) For determination, a pipette was used to transfer 10 ml of measurement solution into a 100‐ml volumetric flask, to which 4 ml of 0.01 mol/L hydrochloric acid and 1 ml of basic lead acetate (add 100 ml of water to 50 g basic lead acetate, allow it to stand still overnight, and filter the supernatant) were added. The solution was diluted to scale with distilled water, mixed well, allowed to stand still overnight, and the supernatant filtered. Twenty‐five milliliters of filtrate was transferred into a 50‐ml volumetric flask, to which 0.1 ml 4.5 mol/L sulfuric acid solution was added. The mixture was diluted with water to scale, mixed well, allowed to stand still overnight, and the supernatant filtered. Using distilled water as a blank reference, the absorbance at 274 nm was measured. The content of caffeine in the hawk tea was calculated using the caffeine standard curve.

The content of amino acids was determined by an amino acid automatic analyzer according to the National Standard of the People's Republic of China GB5009. 124–2016 “Determination of amino acids in foods.” a) The mixed amino acid standard operating solution was prepared to calibrate each amino acid in the hawk tea. Eighteen amino acid standards (accurate to 0.00001 g) were accurately weighed in the same 50‐ml beaker, dissolved in 8.3 ml of 6 mol/L hydrochloric acid solution, transferred to a 250‐ml volumetric flask, diluted to scale with water, and mixed well. One milliliter of the mixed amino acid solution was accurately pipetted into a 10‐ml volumetric flask, to which pH 2.2 sodium citrate buffer solution was added. This was mixed well and used as the standard operating solution. b) The test solution included a certain amount of crushed dry sample (accurate to 0.0001 g) in a hydrolysis tube, to which 10–15 ml of 6 mol/L hydrochloric acid solution was added, followed by 3–4 drops of phenol. The hydrolysis tube was placed into a refrigerant (commercially available salt and ice cubes mixed in a ratio of 1:3 by mass) for 3–5 min, vacuumed and filled with nitrogen three times, sealed, and placed into a hydrolysis furnace at 110 ± 1°C and hydrolyzed for 22 hr. It was then removed, cooled to room temperature, and filtered into a 50‐ml volumetric flask. The hydrolysis tube was washed with a small amount of water several times, following which water was added to scale. One milliliter of filtrate was accurately pipetted into a 15‐ml test tube, dried at 40–50°C by a tube concentrator, and the residue dissolved with 1–2 ml water and then dried under reduced pressure. One to two milliliters of pH 2.2 sodium citrate buffer solution was added for dissolution, shaken and mixed well, and passed through a 0.22‐μm filter membrane for determination. For the determination of tryptophan, the solution was prepared as follows: A certain amount of crushed dry sample (accurate to 0.0001 g) was accurately weighed in a polytetrafluoroethylene liner, to which 4 mol/L 1.50 ml hydrogen lithium oxide solution was added. The liner was inserted into the hydrolysis glass tube, vacuum‐packed to 7 Pa, sealed, and placed in a hydrolysis furnace at 110 ± 1°C and hydrolyzed for 22 hr. It was then removed, cooled to room temperature, transferred to a 50‐ml volumetric flask with a small amount of sodium citrate buffer solution at pH 4.3 [c(Na^+^) 0.2 mol/L], and neutralized with 6 mol/L hydrochloric acid solution. The volume was made up with the above buffer solution and passed through a 0.22 μm filter membrane for determination. For the determination of cystine, the solution was prepared as follows: The dried tea sample was crushed and sieved through a 40‐mesh sieve. A sample not exceeding 75 mg (accurate to 0.0001 g) was placed in a 20‐ml concentration bottle and cooled in an ice‐water bath for 30 min, to which 2 ml of cool performic acid solution [30% hydrogen peroxide and 88% formic acid mixed at 1:9 (V/V), placed at room temperature for 1 hr, placed in an ice‐water bath, and cooled for 30 min] was added, ensuring that the sample was completely moist. The sample was then sealed and placed in a 0°C refrigerator with an ice bath and reacted for 16 hr. We then added 0.5 ml of biased sodium sulfite solution (33.6 g of biased sodium sulfite, water added to 100 ml). The mixture was shaken well, and 17.5 ml of 6.8 mol/L hydrochloric acid solution was added and the sample hydrolyzed at 110 ± 3°C for 22–24 hr. The hydrolysis tube was removed and cooled, and the contents were transferred to a 50‐ml volumetric flask with water and neutralized with 7.5 mol/L sodium hydroxide solution to pH 2.2. Sodium citrate buffer (pH 2.2, 0.2 mol/L Na) was then added to a constant volume, centrifuged, and the supernatant removed for instrument measurement. 3) For determination, a mixed amino acid standard working solution and sample measurement solution were injected into the amino acid analyzer in the same volume, and the concentration of amino acids in the sample was calculated by calculating the peak area with the external standard method. The minimum detection limit of the various amino acids was 10 pmol.

The content of mineral elements was determined by inductively coupled plasma‐mass spectrometry (ICP‐MS) with microwave‐assisted digestion pretreatment as described by Xin et al. (2010) with some modifications, and As and Hg were determined using an AFS‐230E atomic fluorescence spectrophotometer as described by Gao et al. (2018) with some modifications. Crushed dry tea (0.5 g) was placed into the electrothermal digestion tube, to which 8 ml nitric acid was added and the mixture stirred at room temperature for 2 hr. The mixture was then heated to 130°C for 2 hr and then 145°C for 2 hr until it became clear and transparent. It was then cooled to room temperature and transferred into a 50‐ml volumetric flask and shaken well. Five parallel samples were made for each sample, and the average value was used. The blank solution was tested at the same time. The linear ranges for Ca and Mg were from 0 to 100 μg/L; those for Fe, Zn, and Mn were from 0 to 10 μg/L; those for Cu and Pb were from 0 to 5 μg/L; and those for Se, Ni, and Cr were from 0 to 0.5 μg/L. As and Hg were determined using an AFS‐230E atomic fluorescence spectrophotometer as described by Gao et al. (2018) with some modifications. For these, 0.5 g crushed dry tea was placed into an electrothermal digestion tube, to which 9 ml nitric acid and 1 ml perchloric acid were added, stirred at room temperature for 2 hr, and then heated up to 130°C for 2 hr and 145°C for 2 hr until the solution became transparent. The mixture was then cooled to room temperature, 10 ml of water was added, and 10% sodium hydroxide solution was added to neutralize until phenolphthalein discoloration. Two milliliters hydrochloric acid, 2.5 ml 5% thiourea, and 5% ascorbic acid mixed solution were added, and the mixture was transferred into a 25‐ml volumetric flask and shaken well. Five parallel samples were measured for each sample, and the average value was used. The blank solution was measured at the same time. The linear ranges for As and Hg were from 0 to 0.5 μg/L.

#### 2.2.3 Antioxidant activity

##### DPPH• scavenging activity

The DPPH• scavenging activity was determined as described previously (Wan et al., 2013). An acid methanolic solution of DPPH (1,1‐diphenyl‐2‐picrylhydrazyl) was used to assess the antioxidant activity of the hawk tea sample. Trolox was used as the positive control. A 2‐ml sample was added to a methanolic solution of DPPH (2.0 × 10^−4^ mol/L, 2.0 ml) and 5 ml of methanol. The mixture was then vigorously shaken for 10 s and left to stand at room temperature for 30 min. The scavenging activity of the hawk tea on DPPH• was determined by the absorbance at 517 nm, and the percentage of scavenging activity was calculated according to the following equation:

$$\text{Scavenging activity (}\%)=\left({\text{Abs}}_{\text{control}}‐{\text{Abs}}_{\text{sample}}\right)/{\text{Abs}}_{\text{control}}\times 100,$$

where Ab_scontrol_ is the absorbance of the control (without the test sample), and Abs_sample_ is the absorbance of the sample (with the test sample). The results were expressed as the EC_50_ values (µg/ml) for the 50% DPPH• scavenging effect concentration.

##### Ferric reducing activity power assay

Ferric reducing activity power (FRAP) was evaluated according to the previous report using FeSO_4_⋅7H_2_O as the standard (Frank et al., 2012). The fresh FRAP reagent was prepared before using, which contains 25 ml acetate buffer (300 mmol/L, pH 3.6), 2.5 ml TPTZ solutions (10 mmol/L in 40 mmol/L HCl), and 2.5 ml of FeCl_3_⋅6H_2_O solution (20 mmol/L). The reagent was warmed to 37°C, and then, 500 µl was placed in a cuvette and the initiate absorbance was read. 20 µl of the sample solutions was added to the cuvette, and the absorption was determined at 593 nm using the spectrophotometer (UV‐1750, Shimadzu). Values were calculated using the FeSO_4_⋅7H_2_O standard curve.

### 2.3 Statistical analysis

All of the analyses were carried out in triplicate, and the results were reported as means ± *SD* (standard deviation). ANOVA and Duncan's tests with α = 0.05 were used to determine the differences between the assays. Differences at *p* < .05 were considered to be significant. All of the statistical analyses were performed with SPSS 18.0 (SPSS Inc., Chicago, US).

## 3 RESULTS AND DISCUSSION

### 3.1 Inclusions

**FIGURE 2:**
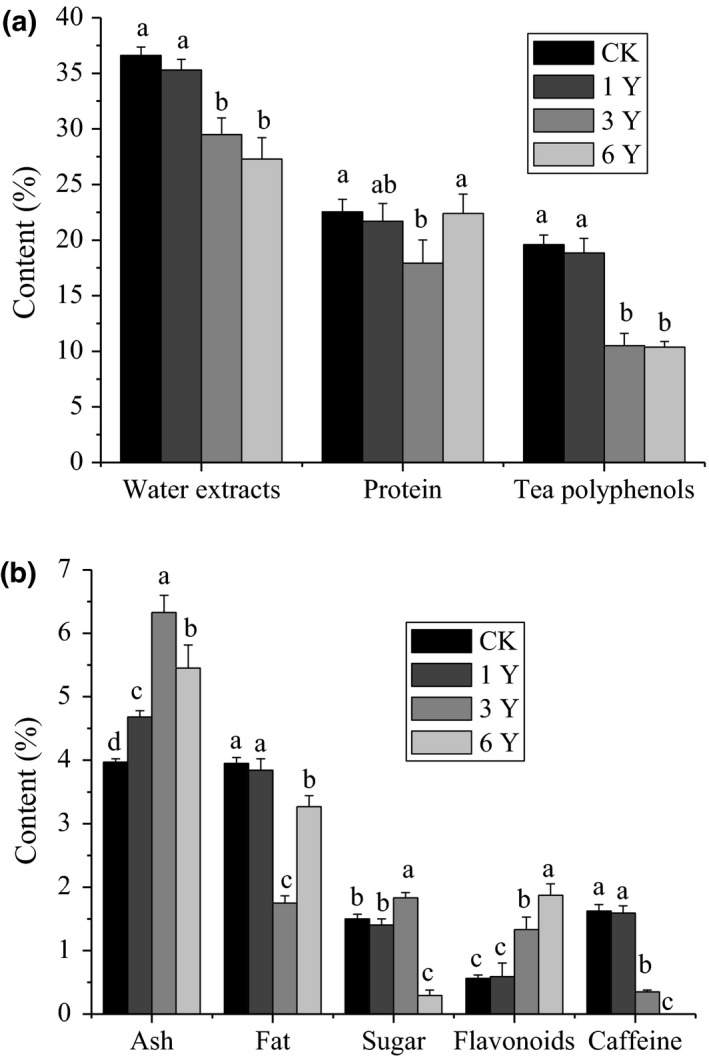
The inclusions content of hawk tea made by boiling water fixation during different storage time (%). CK: the sample stored for 0 year; 1Y: the sample stored for 1 year; 3Y: the sample stored for 3 years; 6Y: the sample stored for 6 years. All of the data are expressed as mean ± standard deviation (*SD*) of three replicates. *Values in each column with different letters are significantly different (*p* < .05)

The inclusions including water extracts, protein, tea polyphenols, total ash, crude fat, soluble sugar, total flavonoids, and caffeine were analyzed. As can be seen from the results in Figure 2, the contents of water extracts and tea polyphenols gradually decreased with increasing storage time (Figure 2a). The water extracts content of tea reflects the amount of soluble substances in tea. A greater water extract value implies a more concentrated tea soup. The soluble extract of tea has traditionally been regarded as an important international standard for tea quality, as it can be directly absorbed and utilized by human body (Harbowy et al., 1997). It is generally required to be more than 20% in the high‐quality green tea. According to the results from Figure 2a, the contents of water extract detected in hawk tea prepared by boiling water fixation method were all higher than 20% during 6 years storage time, which exceeded the content standard of high‐quality green tea. With the prolongation of storage time, the content of water extracts in hawk tea gradually decreased. When stored for 1 year (1Y), 3 years (3Y), and 6 years (6Y), the content of water extract was 87.34%, 75.42%, and 54.27% of the new tea (CK), respectively. The reason for this phenomenon might be that with the extension of storage time, a series of chemical reactions, such as oxidation and hydrolysis, occurred in the main components of tea, which reduced the content of water extract to a certain extent. Similarly, tea polyphenols, one of the important chemical components that determine the quality of tea, exhibited a similar variation pattern to that of water extracts. The content of tea polyphenol was 96.17%, 53.62%, and 52.96% of the new tea (CK) at 1, 3, and 6 years of storage. This was because on the one hand, tea polyphenols would polymerize and oxidize into macromolecular substances, and on the other hand, tea polyphenols would undergo hydrolysis during storage.

Protein is an important material foundation in human life activity organization. Proper intake of protein can not only ensure people's normal life, but also have an important impact on human health. It has been reported that proteins have biological activities such as antitumor, antibacterial, and immunoenhancement (Zhou et al., 2019). In the hawk tea, the protein content is higher (22.53%) and fluctuated up and down with a constant decrease in 3 years storage and then an increase to the same level as that of new tea in the second 3 year storage (Figure 2a). It might be caused by the different rates of protein hydrolysis and decomposition into amino acids, and amino acids conversion and polymerization into proteins. During the first three years of storage, the rate of protein hydrolysis and decomposition was fast, but in the next three years of storage, the rate of amino acid conversion and polymerization was fast.

Total ash is one of the legal chemical inspection items for tea export and is negatively correlated with the quality of tea. The total ash contents of the hawk tea prepared by boiling water fixation fluctuated up and down with a continuous increase from 3.97% to 6.33% for the first 3 year storage (3Y) and then a decrease to 5.45% for the second 3 year storage (6Y). And the storage time had a significant effect (*p* < .05) on the total ash content of hawk tea. The change trend of crude fat content was just opposite to that of total ash content. Its content decrease from 3.95% to 1.75% for the first 3 year storage (3Y) and then an increase to 3.27% for the second 3 year storage (6Y).

Soluble sugar is an important component of tea taste and leads to a unique sweet aftertaste. Generally, dried tea leaves contain approximately 4%–6% soluble sugars (Harbowy et al., 1997). The content of soluble sugar in the hawk tea ranged from 0.29% to 1.83% in 6 years storage time and reached the highest in the third year of storage, but was the lowest in the sixth year of storage. In the first three years of storage, the content of soluble sugars showed an upward trend, which might be attributed to the transformation of insoluble sugar. However, in the next three years of storage, the change was just the opposite, and the decrease was obvious (*p* < .05), which indicated that the insoluble sugar had been completely or little transformed during this period, but the degradation and conversion of soluble sugars were serious. Soluble sugars could be oxidized and fermented, could be transformed into various acidic substances, and could produce maillard reaction with amino acids. The content of total flavonoids increased gradually. When stored for 1 year (1Y), 3 years (3Y), and 6 years (6Y), the content of total flavonoids was 1.05, 2.36, and 3.32 times of the new tea (CK), respectively. Caffeine is a powerful central nervous system and cardiac muscle stimulant that contributes to the unique quality of tea. However, high levels of caffeine can irrigate the gastrointestinal tract and lead to other adverse effects (Gramza‐Michalowska, 2014). Therefore, it is advisable that the caffeine content of tea of reduced. The change of caffeine content detected in hawk tea made by boiling water fixation was decreased significantly from 1.62% to 0%. And in the sixth year of storage, caffeine was not detected in hawk tea.

The above results indicated that the contents of inclusions in hawk tea would change during the storage. Due to the influence of the external environment, hawk tea could occur hydrolysis, oxidation, and degradation, especially the effect of temperature and humidity. And the contents of inclusions in hawk tea changed less within 12 months.

### 3.2 Amino acids

Amino acids are the main substance of tea and are closely related to the aromatic properties of the tea. They also have an obvious influence on the taste and color of the tea soup, and the content of free amino acids in tea is positively related to tea quality (Harbowy et al., 1997). Eighteen types of amino acids were detected in the hawk tea prepared by boiling water fixation, and their contents are shown in Table 1. Overall, the total contents of 8 types of essential amino acids and 18 types of amino acids in hawk tea presented a trend of descending first, then increasing, and then decreasing with storage time and had significant differences (*p* < .05).

**TABLE 1:** The amino acids content of hawk tea made by boiling water fixation during different storage time (mg/100 g)

Amino acid	CK	1Y	3Y	6Y
Lysine^#^	1,215.00 ± 7.37a	1,071.75 ± 5.94a	557.50 ± 1.70b	51.00 ± 1.84c
Threonine^#^	895.00 ± 6.66b	858.25 ± 1.51b	2,787.50 ± 5.52a	55.25 ± 1.24c
Valine^#^	2,431.00 ± 12.66a	2,320.00 ± 4.24a	517.50 ± 2.40b	32.75 ± 1.56c
Isoleucine^#^	1779.00 ± 5.51a	1676.75 ± 4.45a	302.50 ± 0.85b	48.00 ± 2.36c
Leucine^#^	2,449.00 ± 9.85b	2,154.25 ± 4.94b	10,750.00 ± 12.73a	72.75 ± 1.51c
Methionine^#^	225.00 ± 7.02b	155.00 ± 2.81c	840.00 ± 2.80a	10.75 ± 0.51d
Histidine^#^	799.00 ± 6.24b	872.00 ± 5.65a	570.00 ± 2.83c	100.75 ± 1.59d
Phenylalanine^#^	771.00 ± 9.07a	785.00 ± 4.24a	740.00 ± 4.01b	43.00 ± 2.82c
Arginine	0.00 ± 0.00c	0.00 ± 0.00c	332.50 ± 2.97a	110.00 ± 1.69b
Alanine	1617.00 ± 4.73b	2,138.75 ± 7.74a	765.00 ± 5.93c	104.50 ± 1.27d
Glycine	1819.00 ± 8.02a	1796.00 ± 4.24a	135.00 ± 2.82b	0.00 ± 0.00c
Serine	1,031.00 ± 4.93b	984.25 ± 2.24b	7,820.00 ± 15.55a	39.25 ± 3.26c
Proline	3,102.00 ± 5.57a	2,803.75 ± 3.95b	275.00 ± 1.41c	47.25 ± 1.30d
Glutamic acid	829.00 ± 3.51b	966.25 ± 2.70b	2,942.50 ± 3.81a	0.00 ± 0.00c
Aspartic acid	786.00 ± 7.55b	855.75 ± 2.59b	1,382.50 ± 8.34a	59.50 ± 1.97c
Cystine	110.00 ± 5.03b	112.00 ± 4.24b	642.50 ± 6.78a	78.25 ± 1.24c
Tyrosine	517.00 ± 6.03a	583.00 ± 4.24a	80.50 ± 1.83b	31.50 ± 1.13c
Tryptophan	0.00 ± 0.00b	0.00 ± 0.00b	0.00 ± 0.00b	23.50 ± 1.27a
Essential amino acids	10,564.00 ± 10.07b	9,839.00 ± 8.97c	17,065.00 ± 17.27a	414.25 ± 5.05d
Total amino acids	20,375.00 ± 19.57b	20,078.75 ± 18.99c	31,440.50 ± 30.35a	908.00 ± 10.04d

Note

All of the data are expressed as mean ± standard deviation (*SD*) of three replicates.

Abbreviations: 1Y, the sample stored for 1 year; 3Y, the sample stored for 3 years; 6Y: the sample stored for 6 years; CK, the sample stored for 0 year.

Values in each column with different letters are significantly different (*p* < .05).

The contents for lysine, valine, isoleucine, glycine, and proline decreased continuously in the storage period of 6 years. Especially for glycine, its content dropped to 0 in the sixth year. The contents for glutamic acid kept growing in the first 3 year storage (3Y), from 829.00 mg/kg to 2,942.50 mg/kg, and then drastically decreased to 0 in the next 3 years storage (6Y). But for tryptophan, its content did not detect in the first 3 years storage and then increased to 23.50 mg/kg in the next 3 years storage. As one of essential amino acids of animal, tryptophan has many biological functions, such as regulation of immune function, digestive function, protein synthesis, and animal stress(Liu et al., 2019). For arginine, its content increased from 0.00 mg/kg to 332.50 mg/kg in the first 3 year storage and then decreased to 110.00 mg/kg in the next 3 year storage.

Amino acids, due to the physical and chemical properties of the amino acids themselves, can be oxidized, degraded, and transformed under certain temperature and humidity conditions. For example, amino acids can undergo decarboxylation and oxidative deamination under certain conditions to produce corresponding aldehyde compounds, such as glycine to formaldehyde, alanine to acetaldehyde, and phenylalanine to phenylacetaldehyde. In addition, amino acids, sugars, and catechins can undergo maillard reaction under suitable conditions. This is consistent with the decreasing trend of tea polyphenols and sugars in hawk tea with the prolongation of storage time in Figure 2. During the second to third year of storage, amino acids increased, which might be related to the decomposition of soluble protein. Overall, our experimental results showed that the total content and types of amino acids in hawk tea made by boiling water fixation changed less within 12 months, reached the maximum in the third year, and then reduced.

### 3.3 3 Mineral elements

Tea contains a variety of mineral elements. These mineral elements play an important role in human metabolism, normal life activities and maintenance of acid–base balance. The content of mineral elements in tea is an important quality indicator that determines the quality and grade of tea to a certain extent (Meng et al., 2020).

The mineral elements in hawk tea were determined. As shown in Table 2, none of the samples had Hg and all of the heavy metals were well below the National Limit Standard (NY 659–2003, GB 2762–2012; Table 2) during 6 years storage time, except for Pb in the new tea (13.80 mg/kg > 5.00 mg/kg), which might be affected by heavy metal pollution of tea raw materials, but the specific reasons and accumulation rules need to be further the study. The total content of trace element first decreased, then increased, and then decreased. Variance analysis showed that the difference in the total content of trace element during the storage process had a significant level (*p* < .05). But for heavy metal, the total content decreased from 73.87 mg/kg to 24.58 mg/kg in the first 3 year storage and then significantly increased to 260.67 mg/kg in the next 3 year storage. And the change showed a significant level (*p* < .05).

**TABLE 2:** The mineral elements content of hawk tea made by boiling water fixation during different storage time (mg/kg)

Mineral elements	CK	1Y	3Y	6Y
Ca^a^	2,754.00 ± 16.97a	2,673.50 ± 6.93a	1,432.59 ± 2.77b	32.03 ± 1.43c
Fe^a^	328.00 ± 4.24c	257.25 ± 2.01d	694.19 ± 2.57a	579.75 ± 2.27b
Se^a^	0.13 ± 0.040b	0.12 ± 0.030b	0.10 ± 0.028b	9.66 ± 0.63a
Zn^a^	146.00 ± 4.24a	106.35 ± 2.30b	62.72 ± 1.88c	62.40 ± 1.56c
Mg^a^	2,215.00 ± 12.73c	2,282.50 ± 3.39c	3,399.24 ± 5.75b	4,040.00 ± 8.48a
Mn^a^	430.00 ± 7.07b	355.00 ± 2.82c	389.66 ± 2.65c	636.00 ± 2.85a
Hg	0.00 ± 0.00	0.00 ± 0.00	0.00 ± 0.00	0.00 ± 0.00
Ni	4.50 ± 0.42b	4.58 ± 0.36b	5.53 ± 0.49b	15.29 ± 2.14a
Cu	54.00 ± 0.71b	41.53 ± 1.21b	17.09 ± 0.77c	95.45 ± 2.30a
As	0.17 ± 0.020b	0.13 ± 0.028c	0.14 ± 0.014c	0.73 ± 0.042a
Cr	1.40 ± 0.28c	1.68 ± 0.084b	1.42 ± 0.098c	3.35 ± 0.12a
Pb	13.80 ± 0.42a	4.85 ± 0.24b	0.40 ± 0.042d	4.19 ± 0.35c
Total trace elements	5,873.13 ± 9.04b	5,674.72 ± 7.35c	5,978.50 ± 10.10a	5,359.84 ± 4.67d
Total heavy metal	73.87 ± 0.35b	52.77 ± 0.64c	24.58 ± 0.49d	119.01 ± 3.22a

Note

All of the data are expressed as mean ± standard deviation (*SD*) of three replicates.

Abbreviations: CK, the sample stored for 0 year; 1Y, the sample stored for 1 year; 3Y, the sample stored for 3 years; 6Y, the sample stored for 6 years.

^a^ Trace elements.

* Values in each column with different letters are significantly different (*p* < .05).

Hawk tea has the characteristics of high Ca, high Fe, high Zn, high Mg, and high Mn. Zn And Fe have important physiological function, nutritional function, and clinical significance. Ca and Mn are important for bones and teeth, Mg and Mn are important for the hematopoietic system, and Mg is important for kidney health. In the new tea (CK), the Ca content was as high as 2,754 mg/kg. With the storage time, the Ca content decreased slowly in the first year storage time and then decreased rapidly in the next 5 years storage time. Ca content declined to about 1.16% throughout 6 years storage time (about 97.08% at the first year). The reason might be that Ca continuously combined with organic compounds during storage. Similar to Ca, Zn content decreased during the storage process, decreased rapidly by 57.04% in the first 3 years storage time, and was reduced by 57.26% throughout the entire storage process. The variance analysis showed that the difference during the first 3 years reached significant levels (*p* < .05). For Fe, its content first decreased, then increased, and then decreased. And the difference during 6 years storage time reached significant levels (*p* < .05). In the new tea (CK), the Mg content was as high as 2,215 mg/kg. With the storage time, the Mg content increased slowly in the first year storage time and then increased rapidly in the next 5 years storage time. Mg content increased by 172.23% throughout 6 years storage time (about 3.05% at the first year). For Mn, its content decreased rapidly by 17.44% in the first year storage time and then increased to 636 mg/kg in the sixth year. For Se, its content changed little during the first 3 years (about 0.12 mg/kg), but increased sharply to 9.66 mg/kg during the next 3 years.

It can be seen from Table 2 that the order of heavy metal elements in hawk tea was Cu > Ni>Pb > Cr>As, which might be related to the pollution degree of its growth environment. In the new tea (CK), the Cu content was 54.00 mg/kg. With the storage time, the Cu content decreased rapidly by 68.35% in the third year after a slow decrease in the first year and then increased to 95.45 mg/kg in the sixth year. The Ni content was 4.50 mg/kg in the CK. With the storage time, the Ni content increased slowly in the first 3 years storage time and then increased rapidly in the next 3 years storage time. In the sixth year, Ni content increased to 15.29 mg/kg. Compared with the new tea, the content of Ni increased by nearly 2.4 times. Pb content decreased from 13.80 mg/kg to 0.40 mg/kg during the first 3 years storage time. And its content increased to 4.19 mg/kg in the next 3 years storage time. Variance analysis showed that the difference in Pb content during the storage process reached a significant level (*p* < .05). For Cr and As, their contents changed little in the first 3 years storage time and then increased rapidly in the next 3 years storage time.

Mineral elements can be oxidized, degraded, and transformed with storage time, due to the physical and chemical properties of the mineral elements themselves and their forms of existence in tea. Our experimental results showed that the content of most of the mineral elements changed less within 12 months, and the content of heavy metal reached the maximum in the sixth years storage, except for Pb.

### 3.4 4 Antioxidant activity

A large number of studies have shown that polyphenols and flavonoids have strong antioxidant properties (Chen et al., 2020; Ji et al., 2020). It was found that the polysaccharide and flavonoid of hawk tea had good antioxidant activity (Chen et al., 2019; Tan et al., 2016; Xiao, et al. 2017). However, it is unclear which kind of substance has a significant correlation with the antioxidant activity of hawk tea. This study explored the correlation between the content of flavonoids and polyphenols and antioxidant activity.

**FIGURE 3:**
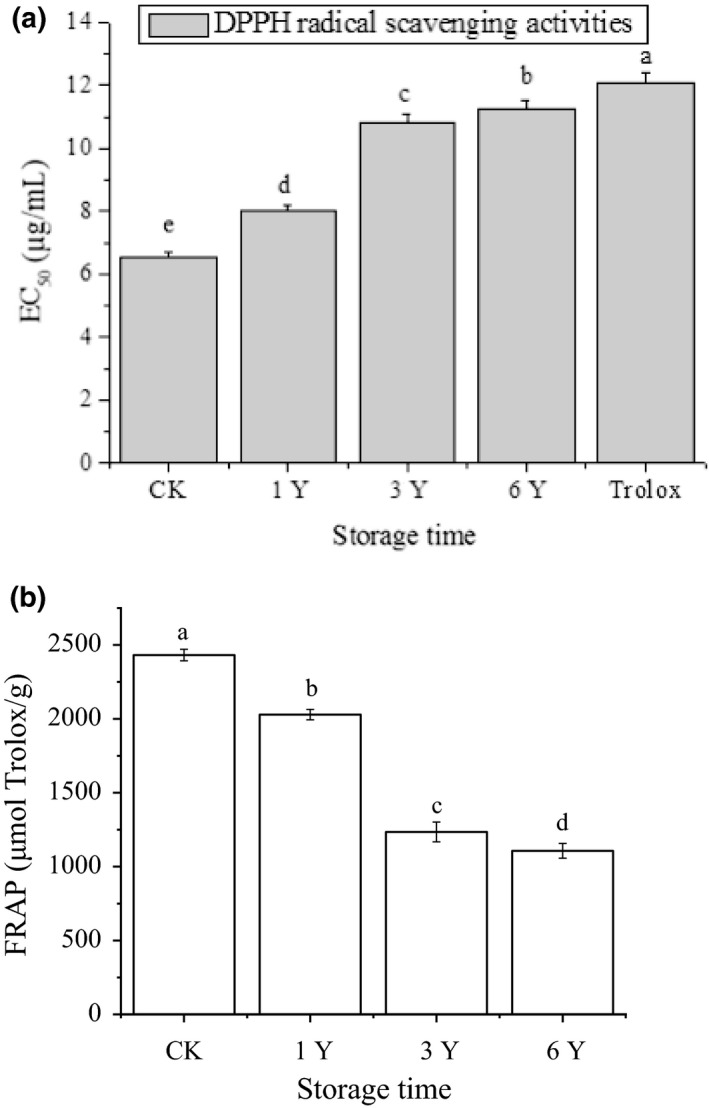
The DPPH radical scavenging activities. All of the data are expressed as mean ± standard deviation (*SD*) of three replicates. Values in each column with different letters are significantly different (*p* < .05)

As shown in Figure 3a, the DPPH radical scavenging activity for all of the hawk tea samples was higher than that of Trolox. The different storage time significantly affected the DPPH antioxidant activity (*p* < .05). Here, a larger EC_50_ implies a lower scavenging activity. With the increase of storage time, the value of EC_50_ increased. The DPPH antioxidant activity was CK > 1Y> 3Y > 6Y. Figure 3b showed that the reducing power of hawk tea samples was negatively correlated with the storage time (*p* < .05). The FRAP values of CK were highest than those of all tested samples, and with the increase of storage time, the FRAP value decreased.

The Pearson correlation among total flavonoids, total polyphenol and DPPH radical scavenging activity, FRAP value were calculated using SPSS software. According to Pearson's significance test, there was a significant negative correlation between the DPPH radical scavenging activity and FRAP value, and the Pearson correlation coefficient was −0.835 and −0.948. However, there was no significant correlation between antioxidant capacity and total flavonoids content of hawk tea stored at different time (*p* > .05). This result indicated that total polyphenols are the main active antioxidant in the hawk tea and have powerful capacity to reduce Fe^3+^ to Fe^2+^_,_ which is consistent with a previous study (Xiao et al., 2015).

## 4 CONCLUSION

This study reported the effect of storage time on inclusions, amino acids, and mineral compositions of hawk tea (*Litsea coreana*) produced by boiling water fixation. With the prolongation of storage time, the biochemical characteristics of hawk tea changed less within 12 months. The total content and types of amino acids in hawk tea reached the maximum in the third year, as well as the content of total trace elements. The contents of water extracts, tea polyphenol, caffeine, lysine, valine, isoleucine, glycine, proline, Ca, and Zn decreased continuously in the storage period of 6 years. And the contents of total flavonoids, Mg, and Ni were just the opposite, showing the trend of increasing year by year. Tryptophan was detected in the sixed year. During the storage period of 6 years, the content of other inclusions, amino acids, and mineral compositions in hawk tea fluctuated up and down.

The antioxidant activity of the new hawk Tea (EC_50_ 6.56 μg/ml for DPPH radical scavenging activity and FRAP value 2,432 Trolox μmol/g) was best, and the activity decreased with the extension of storage time. Total polyphenol is the main antioxidant material in the hawk Tea.

All in all, results from this paper showed that the components of hawk tea have different changes with the extension of storage time. In content, some of the components increased and some of the components decreased. This study provides a theoretical basis for clarifying the relationship between the storage time and the change of the content. At the same time, this study further proves that total polyphenol is the main antioxidant material in the hawk tea.

## CONFLICTS OF INTEREST

The authors declare no conflict of interest.
